# Lessons from a lumbar burst fracture patient infected with SARS-CoV-2

**DOI:** 10.18632/aging.103414

**Published:** 2020-06-22

**Authors:** Shuangqi Yu, Hexing Zhang, Wei Chen, Song Wan, Yi Zhang, Xunzheng Xiong, Fan Ding

**Affiliations:** 1 Department of Spine Surgery, Wuhan Puren Hospital, Wuhan University of Science and Technology, Wuhan 430081, Hubei, China; 2 Department of Orthopedic Trauma, Wuhan Puren Hospital, Wuhan University of Science and Technology, Wuhan 430081, Hubei, China; 3 Graduate School of Wuhan University of Science and Technology, Wuhan 430081, Hubei, China

**Keywords:** SARS-CoV-2, COVID-19, infection, lumbar, fracture

## Abstract

In December 2019, the 2019 novel coronavirus (SARS-CoV-2) began spreading in China. At present, there are no special protocols for treating lumbar burst fracture (LBF) patients infected with SARS-CoV-2. Here, we present our lessons and experiences with a patient presenting with a severe LBF complicated by an occult SARS-CoV-2 infection. The clinical data for a 52-year-old male LBF patient were collected during the incubation period of COVID-19. The patient exhibited no obvious COVID-19-related symptoms prior to his surgery, and his vital signs were stable on the first day after the operation. By postoperative day 3, however, the patient was exhibiting chills and high fever. A chest CT showed a patchy high-density shadow surrounded by ground-glass opacity in the lower portion of his right lung. A nucleic acid test for SARS-CoV-2 was positive, and the patient was then transferred to the Department of Infectious Disease for further special treatment. This case taught that when treating patients with severe trauma within an epicenter of this pandemic, it is crucial for healthcare workers to be vigilant so as to avoid potential widespread outbreaks of COVID-19 within hospitals.

## INTRODUCTION

SARS-CoV-2 refers to a novel coronavirus that firstly reported in Wuhan, Hubei province of China in December 2019 and quickly spread to the world [1]. SARS-CoV-2 belongs to the genus coronavirus, which is able to infect mammals, including humans [2]. The genetic characteristics of SARS-CoV-2 are significantly different from the acute respiratory syndrome coronavirus (SARS-CoV) and the middle east respiratory syndrome coronavirus (MERS-CoV) [3]. A recent study reported that SARS-CoV-2 is very similar to another virus in its family, which is carried by bats, leading some investigators to speculate that bats may host this new virus [4]. Currently, around 3.27 million cases have been confirmed, with 234,000 deaths worldwide [5].

Lumbar burst fracture (LBF) is very common in the elderly, and it is well known that elderly fracture patients are susceptible to pulmonary infection. This is especially true for patients for spinal fractures, which make it difficult for them to walk. This raises the question, what do we do when a fracture patient is infected with SARS-CoV-2? There is currently no protocol for this situation. Here, we summarize our experience and lessons learned when diagnosing and surgically treating a patient with a severe LBF who is also infected with SARS-CoV-2.

## Patient and treatment

**Figure 1:**
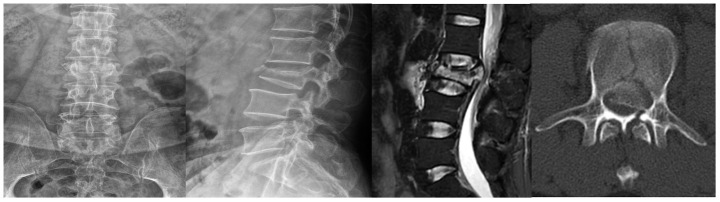
**Lumbar X-rays, MRI, and CT examinations of the patient.**

**Figure 2:**
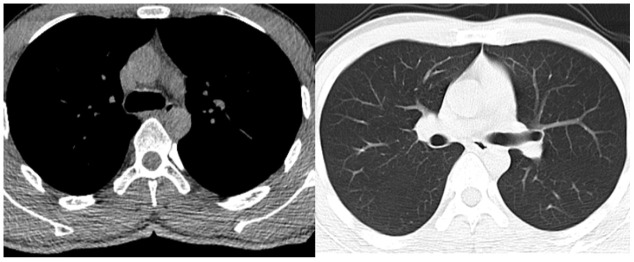
**Preoperative chest CT examination of the patient.**

A 52-year-old male suffering with a L3 LBF presented with lower back pain and serious weakness in both lower limbs. The patient had history of shopping while wearing an ordinary mask. Physical examination indicated that his bilateral hallux dorsal extensor muscle strength level was 2, bilateral tibialis anterior muscle strength was level 2, bilateral quadriceps muscle strength was level 4, and bilateral iliopsoas muscle strength was level. X-ray, magnetic resonance imaging (MRI), and computed tomography (CT) examinations revealed an L3 LBF as well as severe spinal cord compression (Figure 1). The patient denied experiencing fever, cough, sputum production, dyspnea, nausea or vomiting. There was no significant lung abnormality on preoperative CT examination (Figure 2), and tests for viruses indicated the patient to be influenza a virus RNA (-), influenza b virus RNA (-), and respiratory syncytial virus RNA (-). Routine blood counts showed leucocytes 11.43 g/L, erythrocytes 4.87 g/L, hemoglobin 139 g/L, platelets 208 g/L, neutrophils 88.5%, lymphocytes 5.9%, monocytes 5.5%, eosinophils 0%, and basophils 0.1%.

**Figure 3:**
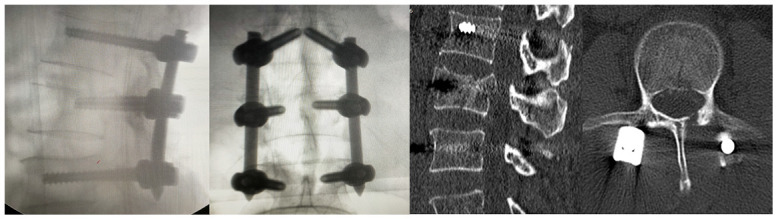
**Postoperative X-rays and CT examination of the patient.**

Lumbar posterior decompression and fixation was performed in a laminar flow, negative pressure operating room. All procedures were performed while strictly adhering to biosafety level 3 standards. In addition to routine personal protection, goggles were worn and postoperative disinfection was performed. The surgery took around 1 hour and 30 minutes, and there were 200 ml of blood loss without blood transfusion. After the surgery, the patient was transferred to the intensive care unit for medical observation and treatment. On the second postoperative day, the patient was safely returned back to the ward with a lumbar brace. Postoperative radiography and CT revealed that the internal repair was well positioned (Figure 3).

**Figure 4:**
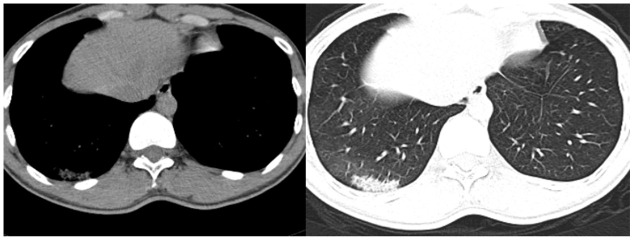
**Chest CT examination of the patient on postoperative day 3.**

On the first postoperative day, the patient had no obvious cough or sputum production, and his vital signs were stable. By the third postoperative day, however, the patient had developed chills and a high fever, which reached 39.5°C. Routine examination showed that his leukocyte count was 7.7 g/L, hemoglobin 126 g/L, neutrophils 81.3%, lymphocytes 8.9%, monocytes 9.5%, eosinophils 0% and basophils 0.3%. Levels of procalcitonin and c-reactive protein were 0.146 ng/mL and 144.9 mg/L, respectively. A chest CT examination showed a patchy high-density shadow surrounded by a ground-glass opacity in the lower portion of the right lung (Figure 4). After consultation with specialists from the Department of Infectious Disease, the patient was immediately isolated in a single room to prevent potential widespread infection. By postoperative day 5, the patient’s temperature had decreased to 37.2°C, and his cough had significantly diminished. A nucleic acid test for SARS-CoV-2 was positive, and the patient was transferred to the Department of Infectious Disease for further treatment, which consisted of cefoperazone sulbactam 1.5 g every 12 hours, abidole hydrochloride 0.2 g orally three times a day, and oseltamivir 75 mg orally twice a day. The patient’s vital signs remained stable, and his cough and fever eventually resolved.

## DISCUSSION

A previous study reported that the incubation period for COVID-19 ranges from 1 to 14 days, with a average period from 3 to 7 days [6]. However, a recent retrospective study by the team of Nanshan Zhong, who is credited with detecting SARS in 2003, indicated that the incubation period for COVID-19 could be as long as 24 days, or 10 days longer than previously understood. That study, which has yet to be peer-reviewed, also suggested that patients can infect other people during the incubation period, echoing the findings of other reports about this virus. The most common symptoms at onset of COVID-19 are fever, fatigue, dry cough, myalgia, and dyspnea. Less common symptoms are headache, dizziness, abdominal pain, diarrhea, nausea, and vomiting [7]. The data from a recent study showed that up to 29% of COVID-19 patients are healthcare workers. Indeed, updated data indicate that in China, a total of 1716 healthcare workers were infected by SARS-CoV-2, and 16 of them have died. The adverse effect of infection of healthcare workers on the prevention and treatment of COVID-19 is enormous. On the one hand, loss of workers due to hospital-related transmission and infection exacerbates the existing shortage of healthcare workers. On the other hand, widespread infection of healthcare workers increases the public’s fear of COVID-19, which is harmful to social stability.

Fever occasionally develops after surgery as a stress response to the procedure [8–9]. In the present case, the elevated neutrophils and the reduction in lymphocytes indicated the possibility of infection, though the chest CT revealed no obvious abnormity. However, by the time the patient developed fever with an obvious dry cough on the day postoperative day 5, the chest CT definitely showed signs of pneumonia, and a nucleic acid test confirmed the SARS-CoV-2 infection on postoperative day 7. The symptoms were initially occult in this case. This patient had no significant symptoms of pulmonary infection at admission, and the chest CT results also did not support a diagnosis of pulmonary infection. Nonetheless, because of the ongoing COVID-19 epidemic, we decided to perform the spinal surgery in a laminar flow, negative pressure operating room. All procedures were performed strictly according biosafety level 3 standards, which are vital for avoiding widespread outbreak of COVID-19. Moreover, to avoid potential cross-infection, all the healthcare workers involved in this case self-isolated for 14 days. This including 3 surgeons, 1 anesthesiologist, 2 operating room nurses, and 2 ward nurses. Fortunately, no one was infected by this patient.

Several lessons can be learned from this case. First, for emergency surgery, comprehensive preoperative examinations should be performed to exclude the possibility of SARS-CoV-2 infection, and use of laminar flow, negative pressure operating rooms is strongly recommended during this COVID-19 pandemic. Second, the incubation period of COVID-19 should be given full attention, even for patients with negative results on the routine tests, perioperative isolation measures should be strong enough to prevent cross-infection among healthcare workers. Finally, for patients with confirmed SARS-CoV-2 infections, consultation with infectious disease specialists should be completed in a timely manner, and the necessary reports should be produced and submitted. If available, patients should be transferred to designated or specialized departments or hospitals for further diagnosis and treatment when necessary.

## CONCLUSION

To reduce the possibility of infection of healthcare workers and to avoid potential widespread infection in hospitals, healthcare workers must take the incubation period of COVID-19 into consideration and be highly vigilant when treating emergency patients during this pandemic.

### Ethical standards

All procedures performed in studies involving human participants were in accordance with the ethical standards of the institutional and/or national research committee (Puren Hospital, Wuhan University of Science and Technology, reference number 3-2020-0131) and with the 1964 Helsinki declaration and its later amendments or comparable ethical standards.
